# Robertsonian and Balanced Reciprocal Translocation in Both Child and Mother with a History of Recurrent Abortions

**Published:** 2020

**Authors:** Subhadra Poornima, Swarnalatha Daram, Rama Krishna, Qurratulain Hasan

**Affiliations:** 1- Department of Genetics and Molecular Medicine, Kamineni Hospitals, L.B. Nagar, Hyderabad, India; 2- Department of Genetics and Molecular Medicine, Kamineni Life Sciences, Moulali, Hyderabad, India; 3- Department of Biochemistry, Kamineni Academy of Medical Sciences and Research Centre, LB Nagar, Hyderabad, India

**Keywords:** Counselling, Culturing, Reciprocal translocation, Robertsonian translocation

## Abstract

**Background:**

Similar rare Robertsonian and balanced reciprocal translocation in both child and mother with a history of multiple miscarriages in the first trimester was the motive to write this case report. Cytogenetic analysis helps in genetic counselling of infertility, BOH and dysmorphology which in turn helps in pre implantation genetic testing. Although many case reports have already been published about Robertsonian and balanced translocations, this is the first case report in India which showed both types of translocation in the same patient, rob (13;14) and t (4;7). Interestingly, in the same patient, same translocations were also identified in the mother and father having no chromosomal abnormalities.

**Case Presentation:**

Proband with dysmorphology was refered first for karyotyping and later parental karyotyping was performed.

**Conclusion:**

Cytogenetic analysis plays an important role in the diagnosis and management of disease along with prenatal screening.

## Introduction

There are different types of chromosomal abnormalities; however, Robertsonian and balanced reciprocal translocations were identified to have the highest prevalence when compared with other chromosomal abnormalities.

Robertsonian translocations are one of the structural chromosomal abnormalities that occur when the p-arm of two acrocentric chromosomes (13, 14, 15, 21, 22) is lost which combines with the qarm of centromere (1). Previous studies indicated that there is an increased risk of infertility, spontaneous abortions or chromosomally unbalanced offspring for carriers of Robertsonian translocation (2, 3).

Balanced reciprocal translocations are also the structural chromosomal abnormality where there is no loss in two non homologous chromosomes, and it occurs with the displacement of the end regions of the chromosomes (3, 4). When balanced reciprocal translocation displacement of the end regions of chromosomes occurs, there will be no loss of chromosomal material between the two non homologous chromosomes (3). Miscarriage is influenced by the size and the genetic content of the rearranged chromosomal fragments (5).

Evaluation of chromosomal abnormalities in parents is useful to explain the cause of miscarriage, as well as the risk associated with future miscarriages, availability or the scope of prenatal diagnosis and testing in the future pregnancies also it provides information for members of extended families who are at the risk of or appropriate for undergoing chromosomal testing.

## Case Presentation

**Figure 1.**
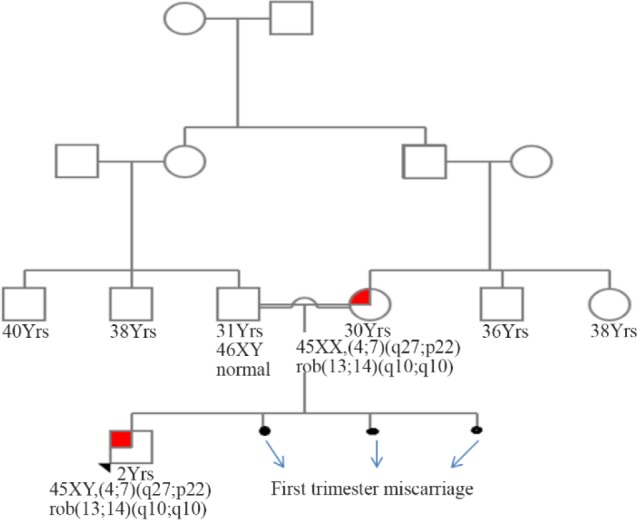
The family pedigree

The proband who is a 2 month male baby with birth weight of 2.8 *Kgs* born to a consanguineous couple, was referred to our comprehensive Genetic Counseling and Diagnostic centre at Hyderabad with the history of facial dysmorphology. Complete clinical, medical and family history along with pedigree was documented with the consent of couple (Figure 1).

It was found to be normal vaginal delivery, full term with no other complications like preeclampsia, GDM during the pregnancy. It was a spontaneous conception without usage of any medication. Ethnicity of patients found to be from Southern part of India.

Parents were healthy and the history indicated they had three miscarriages earlier in the first trimester. There was no evidence of food or drug allergy or alcohol abuse or radiation and chemical exposure. Family history indicated no similar history of miscarriages. In view of history trios (Mother, father and child), cytogenetic analysis was recommended.

### Cytogenetic analysis:

Chromosomal studies were performed from short term phytohaemoagglutinin (PHA) stimulated lymphocytes based on standard methods *ie*. AGT Cytogenetic Laboratory Manual procedures (6). Cytogenetic analysis was performed by GTG-banding at approximately 500 band levels. Chromosomal abnormalities were reported as per the International System for Human Cytogenetic Nomenclature (ISCN, 2016).

## Results

**Figure 2.**
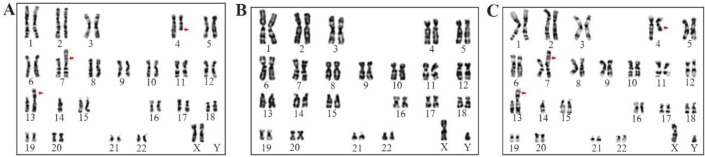
G-banded karyotype images of (A) Mother -45 XX, t (4;7) (q27;p22) rob (13;14) (q10;q10) (B) Father -46 XY Normal (C) Proband (child) -45 XY, t (4;7) (q27;p22) rob (13;14) (q10;q10)

Cytogenetic analysis of proband revealed the presence of a balanced reciprocal translocation between the chromosomes 4, 7 and also a Robertsonian translocation between the chromosomes 13,14: 45 XY, t(4;7) (q27;p22) rob (13;14) (q10;q10) and the same translocations were also identified in the mother 45 XX, t (4;7) (q27;p22) rob (13;14) (q10;q10). Father karyotype found to be normal: 46 XY (Figure 2).

**Figure 3.**
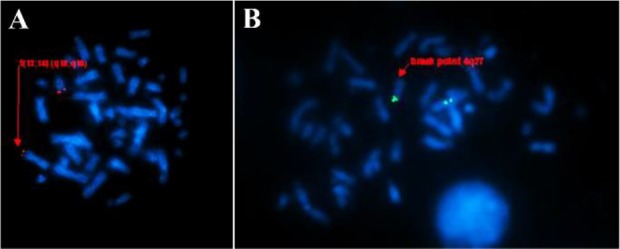
Image showing the Fluorescent in Situ Hybridization on metaphase of proband -45 XY, t (4;7) (q27;p22) rob (13;14) (q10;q10). A) Metaphase image showing the translocation of chromosome 13 and 14, B) Metaphase image showing the breakpoint on chromosome 4 q27

Metaphase FISH was also performed on mother and child sample to reconfirm the breakpoints encompassing the chromosome 13q14 (RB1 probe) and 4p.16 (FGFR3 probe) (Figure 3). FISH probes were purchased from Kreatech, Biotechnology, Amsterdam Netherlands and were used for performing the test and the results revealed the same.

## Discussion

Parental chromosomal rearrangement was first proposed as one of the causes for the recurrent spontaneous miscarriages in 1967. Balanced reciprocal and Robertsonian translocations are found to be one of the most common chromosomal rearrangements. Balanced translocations impede the normal chromosome pairing and segregation at meiosis phase–I, leading to the formation of unbalanced gametes, consequently causing unbalanced abnormal children.

Individuals who are carriers of either a balanced reciprocal or Robertsonian translocation are phenotypically normal; however, there is a probability that they have a significant increased risk of unbalanced gamete production (7).

In the present case, proband had a complex translocation of both a balanced reciprocal translocation and a Robertsonian translocation. The same translocation exists in the mother as well. Robertsonian translocations with chromosomes 13, 14 and 14, 21 have been shown to be the most commonly identified translocations (8). Although the translocations of chromosome 13 and 14 are common, both reciprocal (chr. 4 and 7) and Robertsonian translocation (chr.13 and 14) exist in the same individual and also in the mother. To the best of our knowledge, this is the first case report in Indian population.

Usually the risk will be higher in carriers of double translocations when compared to the single translocation carriers (9). In pregnancies of couple who are carriers of chromosomal translocations, if an embryo with a chromosomal makeup is compatible with life like the monosomy, intrauterine death or a still birth may occur. Unbalanced chromosomal makeup compatible with life may result in congenital anomalies at birth for embryos, *ie*. birth of a balanced translocation carrier of normal phenotype which is the same as parent or birth of a normal child with no chromosomal abnormality.

Prior chromosomal evaluation in couple is even helpful for planning pre implantation diagnosis and testing.

## Conclusion

Cytogenetic analysis for couple prior to planning pregnancy is recommended and in couples with history of recurrent miscarriages and infertility is mandatory. So these facts should be considered and clearly explained to the couple in preconception genetic counselling so that even for carriers of translocations, appropriate Assisted Reproductive Techniques (ARTs) like ICSI (Intra Cytoplasmic Sperm Injection), GIFT (Gamete Intrafallopian Transfer), ZIST (Zygote Intrafallopian Transfer) can be offered based on the requirements. Appropriate testing and the genetic counselling help to control the congenital abnormalities and pave the way to stop the psychological stress or the trauma caused by infertility and bad obstetric history of couples.
